# AbaM Regulates Quorum Sensing, Biofilm Formation, and Virulence in *Acinetobacter baumannii*

**DOI:** 10.1128/JB.00635-20

**Published:** 2021-03-23

**Authors:** Mario López-Martín, Jean-Frédéric Dubern, Morgan R. Alexander, Paul Williams

**Affiliations:** a Biodiscovery Institute, National Biofilms Innovation Centre, School of Life Sciences, University of Nottingham, Nottingham, United Kingdom; b Advanced Materials and Healthcare Technologies, School of Pharmacy, University of Nottingham, Nottingham, United Kingdom; Université de Montréal

**Keywords:** *Acinetobacter*, quorum sensing, *N*-acylhomoserine lactones, RsaM, virulence, biofilm

## Abstract

Acinetobacter baumannii possesses a single divergent *luxR*/*luxRI*-type quorum-sensing (QS) locus named *abaR*/*abaI*. This locus also contains a third gene located between *abaR* and *abaI*, which we term *abaM*, that codes for an uncharacterized member of the RsaM protein family known to regulate *N*-acylhomoserine lactone (AHL)-dependent QS in other beta- and gammaproteobacteria. Here, we show that disruption of *abaM* via a T26 insertion in A. baumannii strain AB5075 resulted in increased production of *N*-(3-hydroxydodecanoyl)-l-homoserine lactone and enhanced surface motility and biofilm formation. In contrast to the wild type and the *abaI*::T26 mutant, the virulence of the *abaM*::T26 mutant was completely attenuated in a Galleria mellonella infection model. Transcriptomic analysis of the *abaM*::T26 mutant revealed that AbaM differentially regulates at least 76 genes, including the *csu* pilus operon and the acinetin 505 lipopeptide biosynthetic operon, that are involved in surface adherence, biofilm formation and virulence. A comparison of the wild type, *abaM*::T26 and *abaI*::T26 transcriptomes, indicates that AbaM regulates ∼21% of the QS regulon including the *csu* operon. Moreover, the QS genes (*abaI* and *abaR*) were among the most upregulated in the *abaM*::T26 mutant. A. baumannii *lux*-based *abaM* reporter gene fusions revealed that *abaM* expression is positively regulated by QS but negatively autoregulated. Overall, the data presented in this work demonstrates that AbaM plays a central role in regulating A. baumannii QS, virulence, surface motility, and biofilm formation.

**IMPORTANCE** Acinetobacter baumannii is a multiantibiotic-resistant pathogen of global health care importance. Understanding Acinetobacter virulence gene regulation could aid the development of novel anti-infective strategies. In A. baumannii, the *abaR* and *abaI* genes that code for the receptor and synthase components of an *N*-acylhomoserine (AHL) lactone-dependent quorum sensing system (QS) are separated by *abaM*. Here, we show that although mutation of *abaM* increased AHL production, surface motility, and biofilm development, it resulted in the attenuation of virulence. AbaM was found to control both QS-dependent and QS-independent genes. The significance of this work lies in the identification of AbaM, an RsaM ortholog known to control virulence in plant pathogens, as a modulator of virulence in a human pathogen.

## Abstract (precis)

Acinetobacter baumannii is a multiantibiotic-resistant pathogen of global health care importance. Understanding Acinetobacter virulence gene regulation could aid the development of novel anti-infective strategies.

## INTRODUCTION

Acinetobacter baumannii is a Gram-negative opportunistic nosocomial pathogen that causes a wide range of infections in humans, most commonly pneumonia but also bacteremia, skin, soft tissue, and urinary tract infections, meningitis, and endocarditis (1). The rise of multidrug-resistant strains has limited the treatment options for this pathogen, which has become a major threat to hospital patients worldwide (2). Indeed, the World Health Organization classified A. baumannii as a critical pathogen for which new antibiotics are urgently required (3). For this reason, a better understanding of the virulence of A. baumannii should aid the development of new therapeutic strategies for preventing and treating Acinetobacter infections. Several virulence factors and regulators involved in A. baumannii pathogenesis have been characterized to date. These include outer membrane proteins (e.g., OmpA), pili, capsular polysaccharide, iron acquisition systems, outer membrane vesicles, secretion systems, and phospholipases (4–9), as well as regulators such as H-NS and two-component systems (10–13). Some A. baumannii strains also undergo phase variation where opaque colony variants exhibit greater motility and virulence but reduced biofilm formation compared to the translucent variants (11). A detailed review of A. baumannii virulence can be found in Morris et al. (14).

One well-established mechanism of virulence gene regulation in diverse pathogens is quorum sensing (QS) (15). This cell-cell communication system is employed by bacteria to coordinate the expression of specific genes as a function of population density. QS is mediated via the synthesis, release, and detection of diffusible signaling molecules such as the *N*-acyl-homoserine lactones (AHLs) (16). A. baumannii and related pathogenic Acinetobacter spp. possess a LuxR/LuxRI QS system consisting of an AHL synthase (AbaI) and a transcriptional regulator (AbaR) that is activated on binding an AHL. Most pathogenic Acinetobacter spp. produce AHLs with acyl side chains of 10 to 12 carbons in length with *N*-(3-hydroxydodecanoyl)-l-homoserine lactone (OHC12) being most commonly encountered. Many strains are however capable of producing other AHLs (17). Several reports have linked QS to biofilm formation and surface motility (18–20), while others have suggested that it plays a role in virulence in a strain and animal model-dependent manner (21, 22). However, our current knowledge of the role of QS in the virulence of pathogenic Acinetobacter spp. is limited.

Located adjacent to the *abaI* gene in Acinetobacter there is an ortholog of the RsaM protein family. These are found in diverse beta- and gammaproteobacteria, including *Burkholderia* spp., Pseudomonas fuscovaginae, Halothiobacillus neapolitanus, and Acidithiobacillus ferrooxidans (23). The first ortholog to be characterized was RsaM in the plant pathogen *P. fuscovaginae*. This was shown to negatively regulate AHL production and was required for full virulence in rice plants (24). Transcriptomic analysis revealed that RsaM partially regulates the QS regulon, as well as modulating the expression of diverse genes in a QS-independent manner (25). Similarly, TofM, the RsaM ortholog found in Burkholderia glumae, represses AHL production while positively regulating toxoflavin and virulence in rice (26). The two RsaM orthologs present in Burkholderia thailandensis are both negatively auto-regulated while being positively controlled by their cognate QS systems (27). In Burkholderia cenocepacia H111, *Bc*RsaM downregulates AHL biosynthesis and modulates swarming motility, biofilm formation, protease and siderophore production (28). Structural and biochemical analysis of *Bc*RsaM showed that it forms dimers in solution and does not appear to bind DNA or AHLs, suggesting that RsaM family proteins act as posttranscriptional or posttranslational regulators (23).

RsaM orthologs clearly play a central role in the regulation of QS-dependent and QS-independent gene expression and virulence in plant pathogens. Here, we investigated the role and regulation of *abaM* in A. baumannii AB5075, a comparatively recently isolated multiantibiotic resistant, hypervirulent strain (29). We show that AbaM, in the opaque variant of A. baumannii 5075, controls AHL production, surface motility, and biofilm formation and is required for virulence in a Galleria mellonella infection model. QS positively regulates *abaM* expression which is turn is negatively autoregulated. Transcriptomic analysis of the *abaM* and *abaI* mutants indicate that the AbaM and QS regulons overlap. These data are consistent with a central role for AbaM in regulating gene expression and the pathobiology of A. baumannii.

## RESULTS

### Organization of the QS locus in *A. baumannii* 5075.

**FIG 1:**
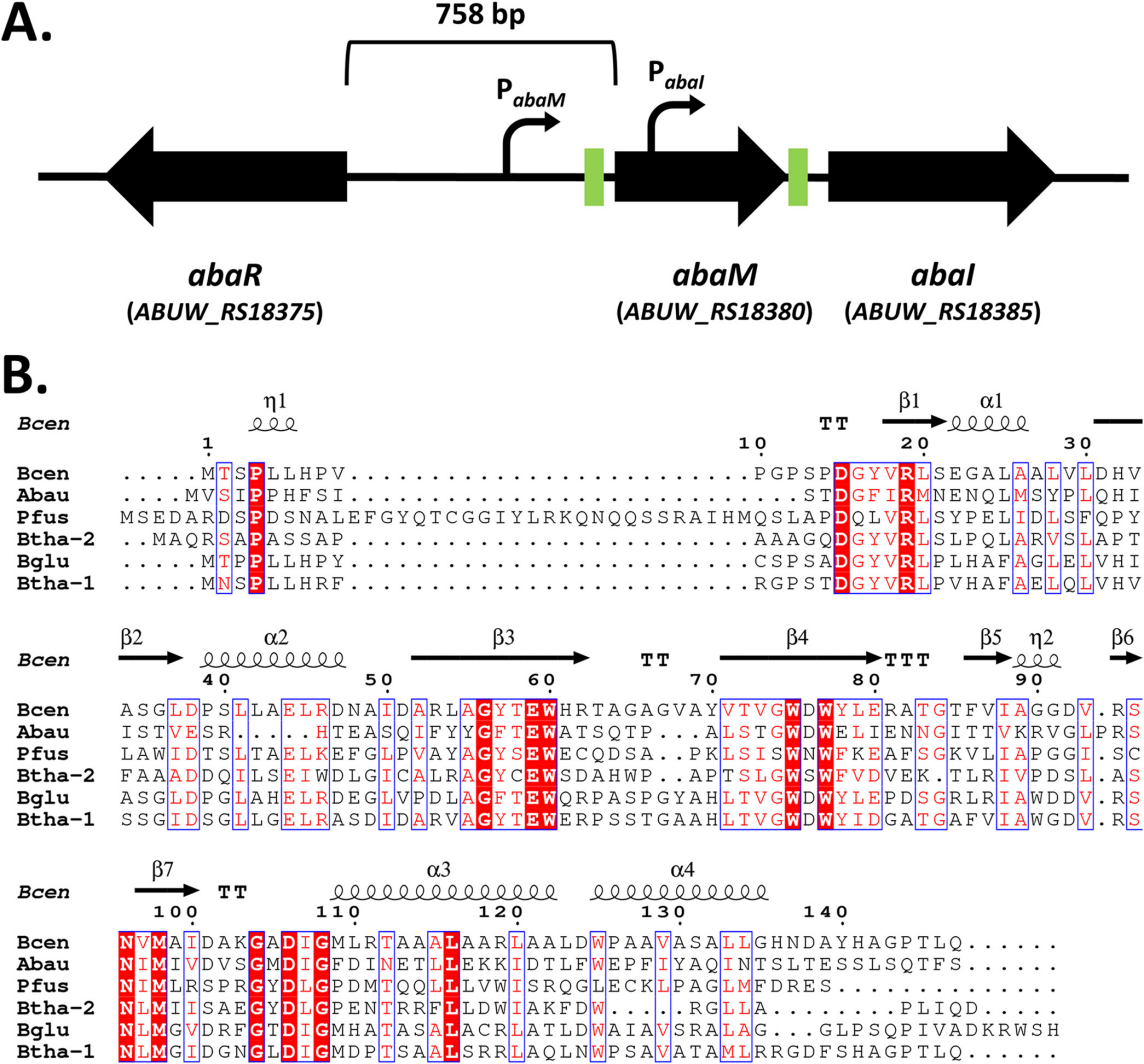
(A) Schematic of the *abaRMI* QS locus in A. baumannii AB5075 showing the organization of the three QS genes and their orientations. Green boxes represent predicted *lux* boxes. Curved arrows represent the predicted *abaM* and *abaI* promoters. (B) Multiple sequence alignment of A. baumannii AB5075 AbaM (Abau) with previously characterized orthologs in other bacterial species: Bcen, Burkholderia cenocepacia J2315 *Bc*RsaM; Pfus, Pseudomonas fuscovaginae UPB0736 RsaM; Btha-2, Burkholderia thailandensis E264 RsaM-2; Bglu, Burkholderia glumae BGR1 TofM; Btha-1, Burkholderia thailandensis E264 RsaM-1. The MUSCLE algorithm (48) was used to create the alignment, and ESPript (49) was used to render residue similarities and generate the final figure. The red background indicates conserved residues. Red residues indicate conservative substitutions. Blue frames indicate highly conserved regions. The secondary structures in B. cenocepacia *Bc*RsaM (PDB entry 4O2H) are displayed above the alignment. η, 3_10_-helix; α, α-helices; β, β-strands; TT, strict β-turns; TTT, strict α-turns.

The genome of A. baumannii AB5075 possesses a single QS locus comprised of two divergently transcribed genes: an AHL synthase gene (*abaI*/*ABUW_3776/ABUW_RS18385*) and a transcriptional regulator gene (*abaR*/*ABUW_3774/ABUW_RS18375*). Between *abaR* and *abaI*, a third gene is located which we term here *abaM* (*ABUW_3775*) (Fig. 1A). The chromosomal organization of these three QS genes is well conserved among Acinetobacter spp (see Fig. S1 in the supplemental material). Despite the location of *abaM* adjacent to *abaI* and transcribed in the same direction, AbaM has only low (ca. 20 to 30%) sequence identity to orthologs present in Pseudomonas fuscovaginae and *Burkholderia* spp. (Fig. 1B). However, it retains the well-conserved regions shared by other RsaM orthologues, including most of the protein secondary structural elements and the characteristic hydrophobic core cluster, consisting of four tryptophan residues (Trp60, Trp75, Trp77, and Trp125) (23).

### AHL production is enhanced in an *abaM* mutant.

**FIG 2:**
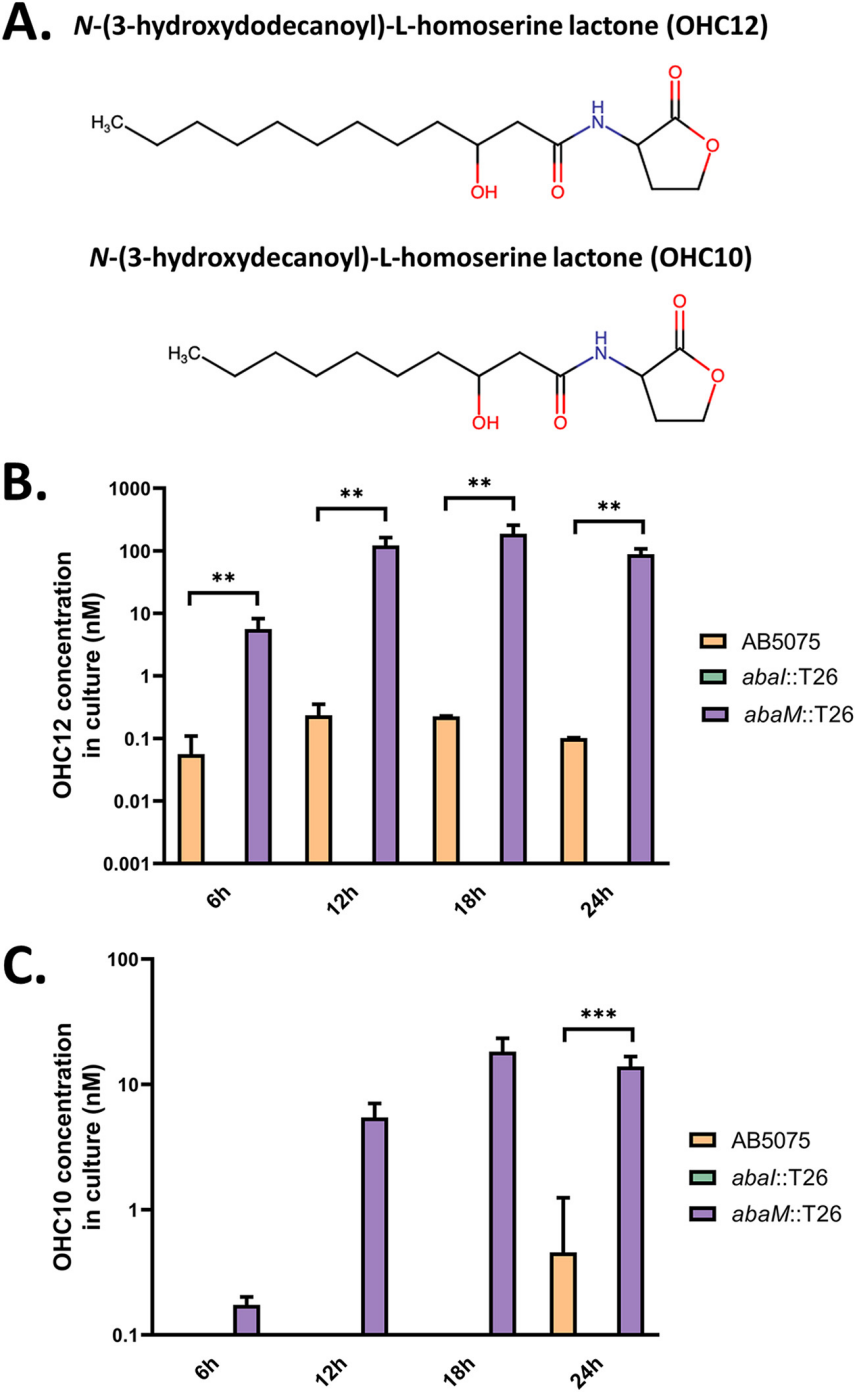
AHL production in wild-type, *abaI*::T26, and *abaM*::T26 strains. (A) Chemical structures of the AHLs produced by A. baumannii AB5075. (B and C) Quantification of OHC12 (B) and OHC10 (C) production throughout growth. Error bars represent the standard deviations between three biological replicates. Asterisks indicate statistically significant differences: **, *P* ≤ 0.01; ***, *P* ≤ 0.001.

AHL production in the opaque variants of the AB5075 wild-type and *abaM*::T26 and *abaI*::T26 mutant strains, respectively, was quantified via liquid chromatography-tandem mass spectrometry (LC-MS/MS) during growth under static conditions, since it appears to be enhanced by surface attachment in other A. baumannii strains (30). OHC12 (Fig. 2A) was the major AHL produced by AB5075 under these conditions. Compared to the wild type, the *abaM*::T26 mutant produced significantly greater amounts of OHC12 at each time point sampled (a difference between 100- and 875-fold) (Fig. 2B). *N*-(3-Hydroxydecanoyl)-l-homoserine (OHC10) was also detected at much lower concentrations in the *abaM*::T26 mutant throughout growth but only in the 24-h sample in the wild type (Fig. 2C). No AHLs were detected in any of the *abaI*::T26 samples (Fig. 2B and C). These data suggest that AbaM is a negative regulator of AHL production.

### Contribution of QS and *abaM* to surface motility, biofilm formation, and virulence.

**FIG 3:**
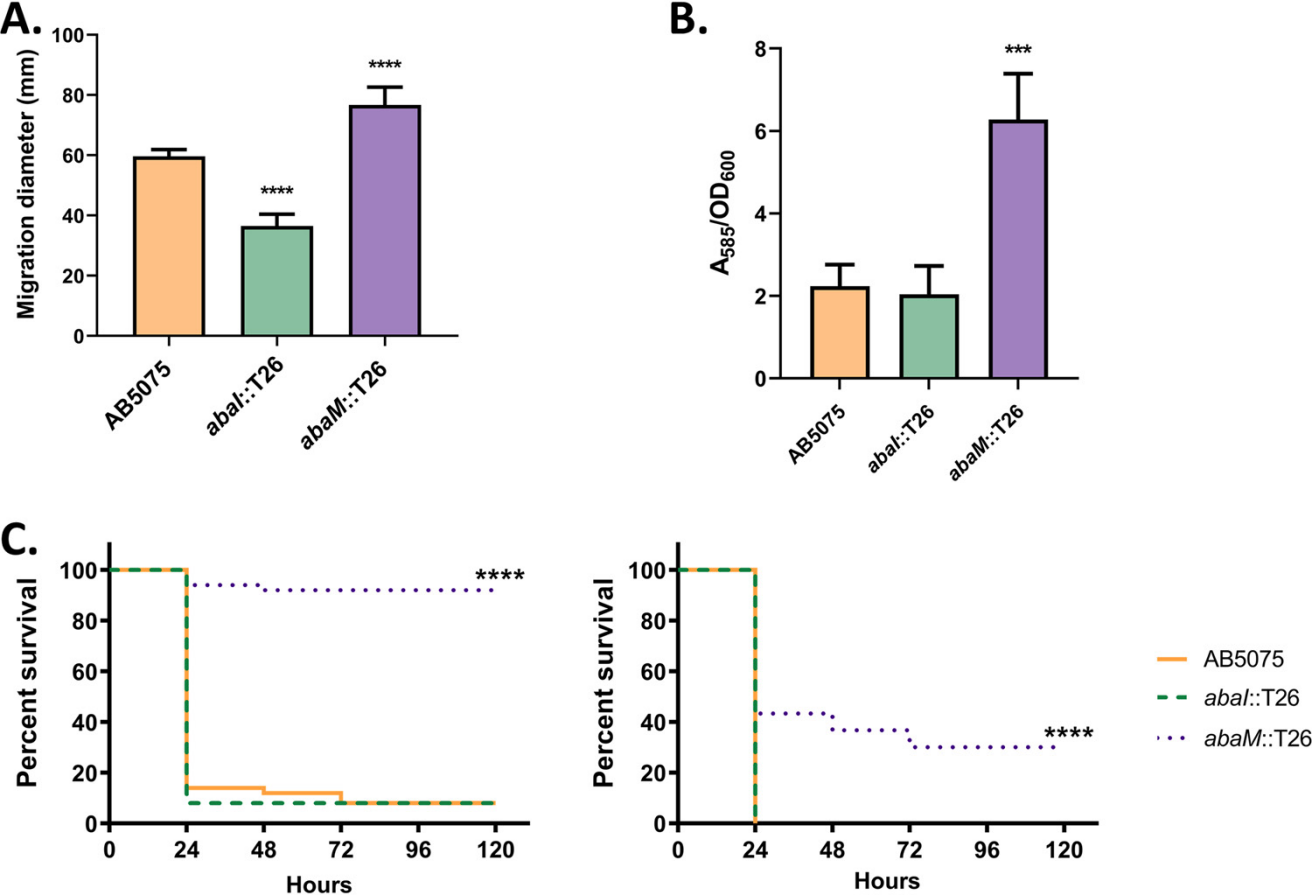
Phenotypic characterization of the Acinetobacter *abaM* and *abaI* mutants. (A) Surface motility on agar. (B) Biofilm formation on polypropylene. For both biofilm and surface motility assays, error bars indicate the standard deviations. Asterisks indicate statistically significant differences compared to the wild-type AB5075 strain. ***, *P* ≤ 0.001; ****, *P* ≤ 0.0001. (C) Galleria mellonella larva killing after inoculation of approximately 2 × 10^4^ (left) or 2 × 10^5^ (right) CFU/larva. Each graph represents data from three independent biological replicates together. At least 30 larvae were used for each strain and assay. None of the control larvae died after 5 days. Asterisks indicate statistically significant differences compared to the wild-type AB5075 strain. ****, *P* ≤ 0.0001.

The surface motility of all three strains on 0.3% Eiken agar LS-LB plates was examined. Compared to the wild type (59.6 ± 0.7 mm), the *abaI*::T26 mutant exhibited significantly reduced surface motility (36.5 ± 1.4 mm), whereas the *abaM*::T26 mutant was significantly more motile (76.7 ± 2.4 mm) (Fig. 3A). The provision of exogenous OHC12 increased the surface motility of both wild-type and *abaI* mutant strains to levels similar to that of the *abaM* mutant (see Fig. S2A).

The ability of AB5075, *abaI*::T26, and *abaM*::T26 strains to attach to abiotic surfaces was evaluated on propylene tubes. The *abaM* mutant formed ∼3-fold more biofilm than did the wild type (Fig. 3B). Under these growth conditions, the biofilm produced by the *abaI* mutant (opaque variant) (Fig. 3B) was not significantly different from the wild type but increased after the exogenous provision of OHC12 (see Fig. S2B).

The contribution of *abaM* and QS to AB5075 virulence was assessed using a G. mellonella larvae infection model (Fig. 3C). No differences in killing were observed between the wild-type and the *abaI*::T26 mutant when injecting either 2 × 10^4^ or 2 × 10^5^ CFU/larva. However, the *abaM*::T26 mutant was significantly less virulent than the parental strain. Larvae injected with 2 × 10^5^ CFU of the *abaM*::T26 mutant also showed a lower survival rate than the larvae injected with 2 × 10^4^ CFU of the wild type or the *abaI*::T26 mutant. Exogenously supplied OHC12 did not affect the virulence of the wild type (see Fig. S2C).

Overall, these results suggest that *abaM* is a negative regulator of surface motility and biofilm formation and required for full virulence in G. mellonella.

### Genetic complementation of the *abaM* mutant phenotypes.

Complementation of the *abaM*::T26 mutant with the *abaM* gene in *trans* (pMQ_*abaM*) restored surface motility (see Fig. S3A) and biofilm formation (see Fig. S3B) and reduced both OHC12 and OHC10 production by approximately 50% (see Fig. S3C and D). However, complementation of the *abaM* mutation did not restore *abaM*::T26 virulence to wild-type levels (see Fig. S3E).

### Transcriptomic analysis of *abaI*::T26 and *abaM*::T26.

To characterize the AbaM and QS regulon of A. baumannii AB5075, we performed transcriptomic profiling of AB5075 in comparison with the *abaM*::T26 and *abaI*::T26 mutants using RNA sequencing (RNA-seq), which was then validated for two key target genes via quantitative real-time PCR. For these analyses, we used total RNA extractions from cells grown for 18 h in static conditions when maximum OHC12 levels are produced by the *abaM* mutant (Fig. 2).

**FIG 4:**
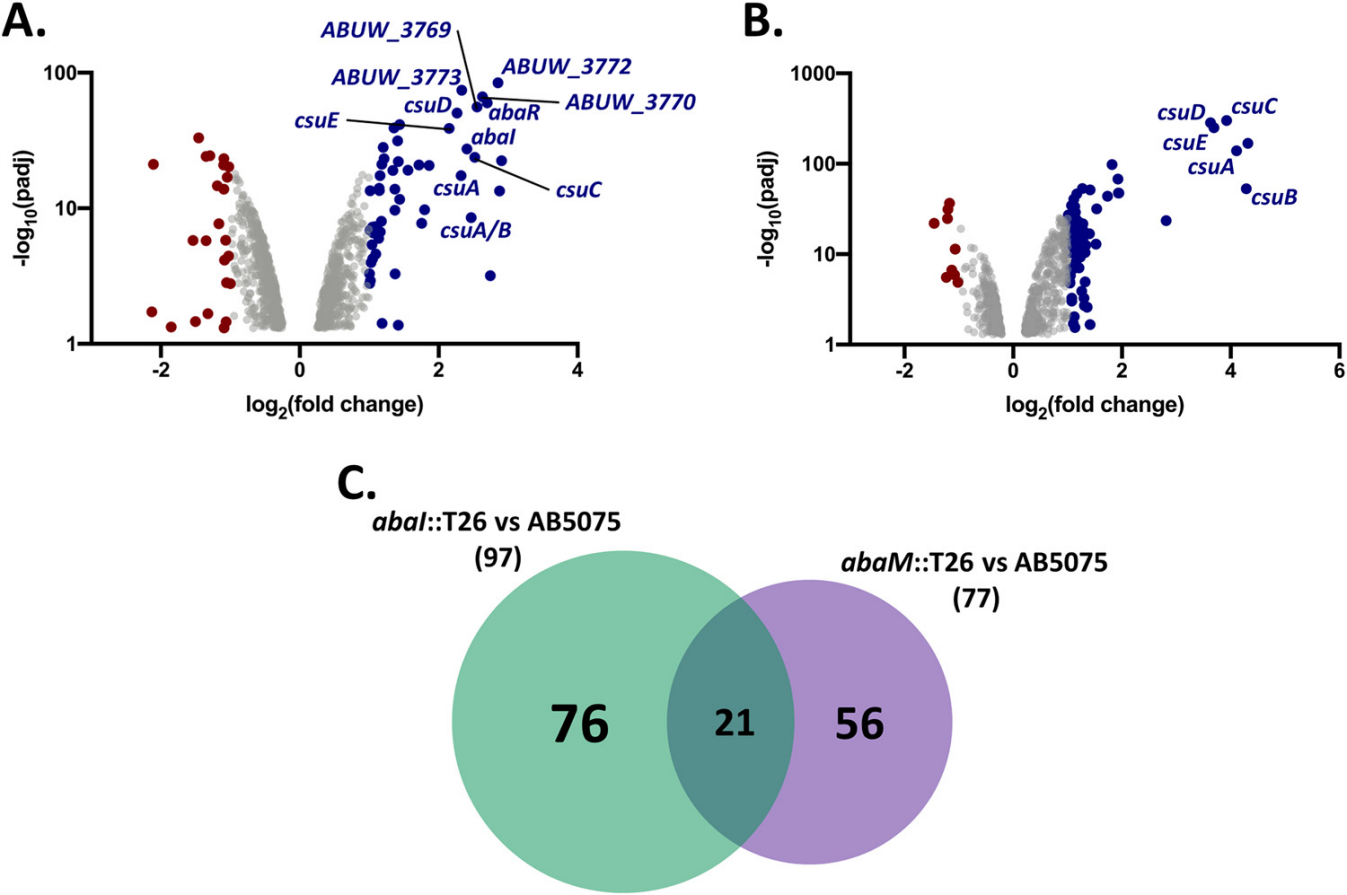
Comparison of the transcriptomes of the *abaI*::T26 and *abaM*::T26 mutants. (A and B) Genes differentially expressed in *abaM*::T26 (A) and *abaI*::T26 (B) strains compared to the wild type. Blue circles indicate upregulated genes, red circles indicate downregulated genes, and gray circles represent genes where changes in expression are unlikely to be biologically significant. (C) Venn diagram showing that AbaM regulates genes that are both QS dependent and QS independent.

Compared to the wild-type strain, 88 genes were upregulated and 9 were downregulated in the *abaI*::T26 mutant, whereas 52 were upregulated and 24 were downregulated in the *abaM*::T26 mutant [log_2_(fold change) ≥ 1] (Fig. 4; see also Tables S3 and S4). Moreover, 21 of the upregulated genes were shared between *abaI*::T26 and *abaM*::T26 (Fig. 4; see also Tables S3 and S4), whereas none of the downregulated genes was coregulated. Among the genes upregulated in both mutants there were all the genes of the *csu* operon, a putative TetR family transcriptional regulator (*ABUW_1486/ABUW_RS07245*) located immediately upstream of the *csu* operon, as well as genes coding for a flavohemoprotein, an uncharacterized transcriptional regulator, a thermonuclease, a sulfate permease, a toxic anion resistance protein, and seven hypothetical proteins (see Tables S3 and S4).

Moreover, some of the genes of the biosynthetic operon involved in the synthesis of acinetin 505, the QS transcriptional regulator *abaR* and the AHL synthase *abaI*, were both upregulated in the *abaM*::T26 mutant, which also differentially expressed genes encoding proteins involved in the stress response, iron acquisition, diverse metabolism and energy production, chaperones, protein folding, and antibiotic resistance, e.g., class D beta-lactamase OXA-23 involved in resistance to carbapenems (see Table S4). Similarly, differentially regulated genes in the *abaI* mutant included diverse metabolic and energy production-related genes, as well as diverse genes coding for transcriptional regulators, stress response-related proteins, and membrane transport proteins (see Table S3).

**FIG 5:**
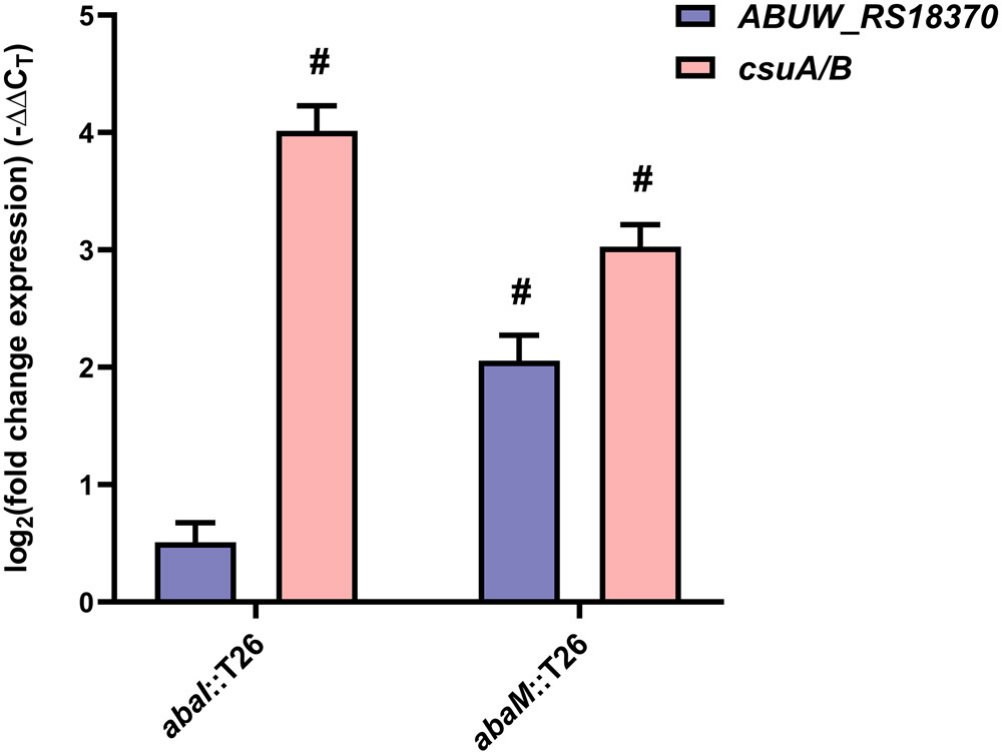
Validation ABUW_3773 and *csu* expression by quantitative real-time PCR. The relative expression of *csuA/B* and *ABUW_3773* in mutants was compared to the wild-type AB5075 strain. The expression was normalized in relation to an endogenous control gene (*rpoB*). Error bars indicate standard deviations between three independent biological replicates. Hashtags indicate a biologically significant difference [|log_2_(fold change)| ≥ 1] compared to the wild type.

To validate the transcriptomic data, qPCR was performed with the same RNA samples used in the RNA-seq for the *csuA/B* and the *ABUW_3773* (the first gene of the acinetin 505 biosynthetic operon) genes (Fig. 5). Compared to the wild type, the data obtained showed a significant increase in *csuA/B* expression in both the *abaI*::T26 and *abaM*::T26 mutants, whereas *ABUW_3773* expression was significantly higher only in the *abaM*::T26 mutant. These results correlate with the data obtained from the RNA-seq experiments.

### Regulation of *abaM*.

**FIG 6:**
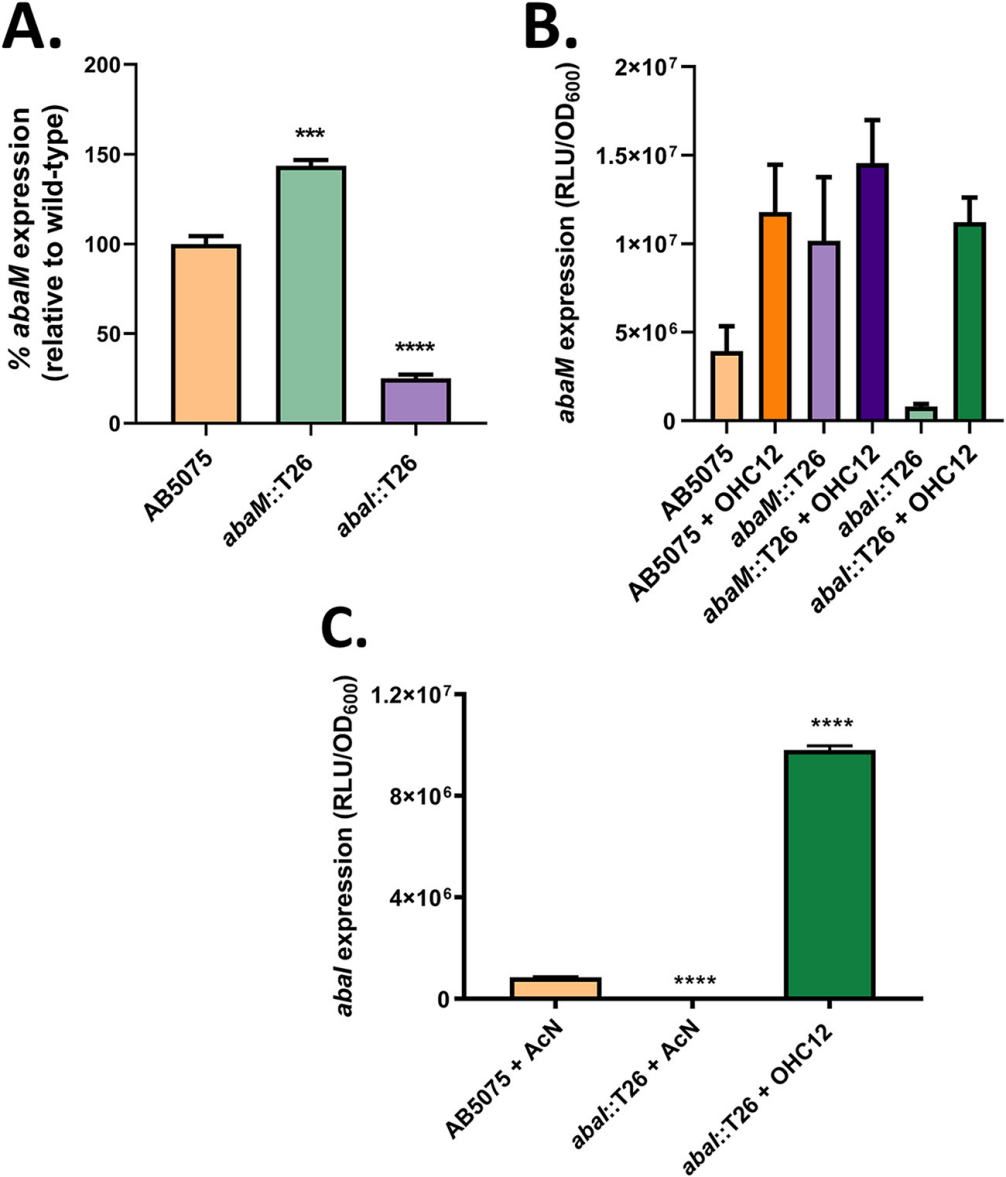
Expression of *abaM* and *abaI*. (A) *abaM* promoter activity in A. baumannii A5075 wild-type, *abaM*::T26, and *abaI*::T26 strains relative to the wild-type strain. (B) *abaM* promoter activity in response to exogenous OHC12 as a function of growth (RLU/OD_600_). (C) *abaI* promoter activity in the wild type and an *abaI* mutant in response to exogenous OHC12 as a function of growth (RLU/OD_600_). Error bars indicate standard deviations between three independent biological replicates. Asterisks indicate statistically significant differences compared to the wild-type AB5075 strain. ***, *P* ≤ 0.001; ****, *P* ≤ 0.0001.

To further elucidate the regulation of *abaM* expression, an *abaM* promoter-*luxCDABE* operon fusion was constructed. This was introduced via a miniTn*7* transposon into AB5075 and both *abaI*::T26 and *abaM*::T26 mutants, and the activity of the predicted promoter was measured by luminescence output in the presence or absence of OHC12. The activity of the *abaM* promoter significantly varied between the strains. In the *abaM*::T26, luminescence was approximately 40% higher than in the wild type, whereas the *abaI*::T26 mutant showed a 75% reduction compared to the parental strain (Fig. 6A). Moreover, exogenous provision of OHC12 increased the *abaM* promoter activity in all three strains (Fig. 6B).

**FIG 7:**
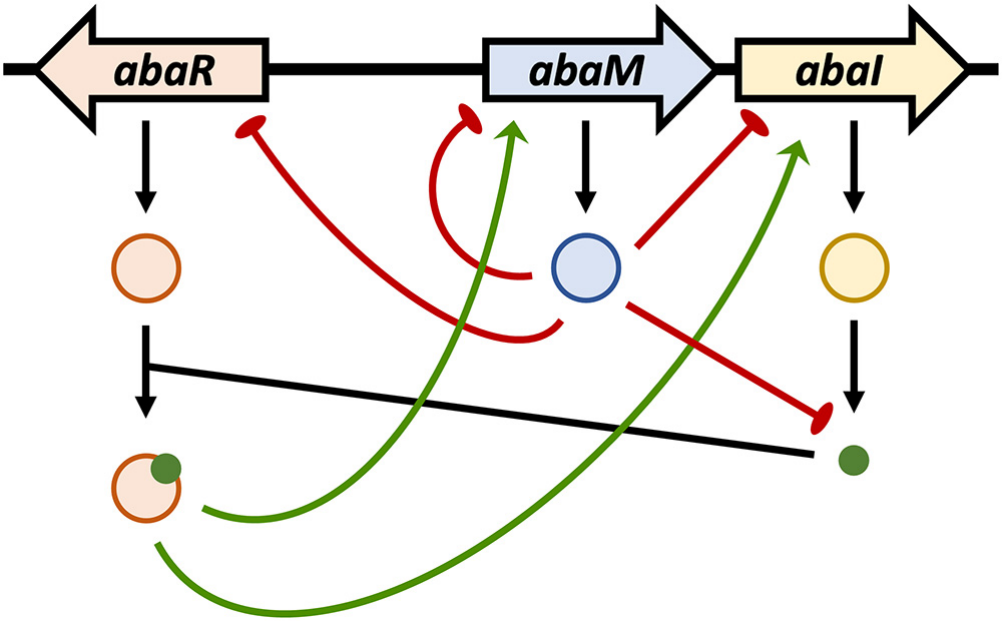
Proposed model for the QS/AbaM IFFL in A. baumannii 5075. AbaR activated by OHC12 positively activates expression of *abaM* and *abaI* and hence OHC12 production. AbaM is negatively autoregulated and also represses expression of both *abaR* and *abaI*. Under the growth conditions used here, AbaM negatively regulates surface motility, biofilm, and Csu pili but is required for virulence as *abaM* mutants are avirulent in Galleria mellonella larvae. Green arrows and red lines represent positive and negative regulation, respectively.

Similarly, Fig. 6C shows that expression of an *abaI*::*lux* promoter fusion, which is reduced in the *abaI* mutant compared to the wild-type strain, is strongly stimulated by OHC12. These data suggest that *abaM* expression is negatively autoregulated but, in common with *abaI*, is positively regulated by QS, which in turn is negatively controlled via AbaM (Fig. 7).

## DISCUSSION

In this study, we have established that the RsaM ortholog, AbaM plays a major role in regulating QS-dependent and QS-independent genes in A. baumannii 5075. Disruption of *abaM* substantially increased AHL production, indicating that AbaM negatively regulates AHL biosynthesis. In A. baumannii 5075, the concentration of OHC12 produced was very low (<1 nM), suggesting that, at least under the static growth conditions used in this study, AbaM exerts tight control over QS. This raises the question of when and under what conditions QS is active in AB5075, especially since the half-maximal responses for LuxR proteins activated by long-chain AHLs is in the 5 to 10 nM range (31). In A. baumannii, the QS locus has an *RXI* topological arrangement previously described in other bacteria, where the *X* gene between the QS regulator (*R*) and the synthase (*I*) genes is a negative regulator of QS. Examples of three different classes of X include *rsaL*, *rsaM*, and *mupX* (32). In this context, AbaM appears to behave similarly to RsaL in the Pseudomonas aeruginosa *lasR/rsaL/lasI* QS system, despite their functional and structural differences. The *rsaL* gene, which is divergent to *lasI*, is positively regulated by LasR and antagonizes the LasR-mediated activation of *lasI*, thus counteracting the QS positive-feedback autoinduction and providing AHL homeostasis (33). This type of regulatory circuit (Fig. 7), termed an incoherent feed forward loop (IFFL) in contrast to simple feed forward loops, can display complex behaviors that include stabilization of output signals and bounded output which ensures robustness against fluctuations in the input signal levels (32, 34). Hence, the large increase in AHL production following deletion of *abaM*, *rsaL*, and *rsaM* genes that results in less-virulent mutants is indicative of the importance of a stabilizing negative regulatory pathway in AHL-dependent QS systems (32, 34).

Consistent with this model, *abaM* expression was found to be negatively autoregulated but positively regulated by QS. *In silico* analysis of the DNA sequence between *abaR* and *abaI* by using Bprom (Softberry) and BDGP (www.fruitfly.org), as well as our RNAseq data, all predict the presence of putative −10 and −35 regions for *abaI* and *abaM*, respectively, suggesting that these genes are not cotranscribed. Similar findings have been reported for *rsaM1* and *rsaM2* from Burkholderia thailandensis (27). Reverse transcriptase-PCR (see Fig. S4 in the supplemental material) confirm that *abaM* and *abaI* in A. baumannii A5075 do not form an operon. Moreover, the *abaM* gene has a predicted *lux* box (CTGGTTAAATATAACAG) 68 bp upstream of the start codon and 178 bp downstream of the −35 and −10 promoter elements. This *lux* box is similar to those found upstream of *abaI* (CTGTAAATTCTTACAG) in both A. baumannii 5075 and A. nosocomialis M2. These results are consistent with an IFFL circuit, although further work will be required to fully characterize its properties and control of genes coregulated by QS and AbaM.

Phenotypic characterization of the A. baumannii *abaM* mutant revealed enhanced surface motility and biofilm formation but reduced virulence compared to the wild type. Previous studies have shown that *rsaM* orthologues are required for full virulence in plants (24, 26), but this is, to the best of our knowledge, the first time that an *rsaM*-like gene has been reported to be required for full virulence in a human pathogen albeit in an insect infection model. The contribution of *Bc*RsaM in B. cenocepacia to swarming motility and biofilm formation has also been reported (28). However, deletion of *Bc*RsaM reduced both swarming and surface attachment, the opposite to that observed for the *abaM* mutant. Interestingly, both biofilm formation and surface motility in Acinetobacter have been associated with increased virulence (10, 35).

Genetic complementation of the *abaM* mutant was achieved for surface motility, biofilm formation, and AHL production but not for virulence in Galleria mellonella. Similar observations have been previously reported for other *abaM* orthologues, most notably *tofM* (26) and *Bc*RsaM (28), leading to the suggestion that RsaM-like proteins may be *cis*-acting regulators (23).

To further define the role of QS in A. baumannii 5075, phenotypic characterization of the AHL synthase mutant, *abaI*::T26, was performed. The mutant did not produce any detectable AHLs, consistent with previous studies and bioinformatic analysis indicating that A. baumannii 5075 possesses a single QS locus that is responsible for AHL production (18, 36). Similarly, disruption of *abaI* negatively affected surface motility and responded to exogenous 3OHC12, as previously noted for other Acinetobacter strains/species (20). In AB5075, biofilm formation in the opaque variant of the *abaI*::T26 mutant was not significantly different from with the wild type. However, it increased well above the wild type in response to exogenous OHC12, consistent with other work on the Acinetobacter AHL synthase (18, 37). Previous studies on QS and biofilm formation in Acinetobacter have been performed with strains that were, in contrast to AB5075, either not phase variable or not known to be phase variable. Since the experiments performed in this study were all carried out with the opaque Acinetobacter variant, we also investigated biofilm formation by the translucent variant. Figure S5 shows that the *abaI* translucent variant produced less biofilm than the wild type. However, biofilm formation increased for both opaque and translucent *abaI* variants in response to exogenous OHC12. Furthermore, our results suggest that QS does not play an important role in virulence in the G. mellonella. While this is not unprecedented (38), the role of Acinetobacter QS in virulence is still not well defined, and other studies suggest that QS may play an important role, depending on the strain and infection model used (21, 22). Overall, our data suggest that for strain AB5075 QS is important in surface motility and biofilm formation but not virulence. However, further work is required to fully elucidate the role of QS in the pathogenesis of Acinetobacter infection and any cross talk with other regulatory networks.

Here, we performed RNA-seq on A. baumannii AB5075 grown in static conditions where AHL production was elevated in order to identify genes regulated via AbaM and QS and likely to be involved in surface attachment and biofilm formation. A comparison of the genes differentially expressed in the *abaM* and *abaI* mutants with the wild type revealed that AbaM has both QS-dependent (∼22% of the QS regulon) and QS-independent gene targets. A similar overlap has been noted for the RsaM regulon and its cognate QS system in *P. fuscovaginae* (25). Among the most upregulated genes in both *abaI* and *abaM* mutants compared to the wild type were those belonging to the *csu* operon (*ABUW_1487-ABUW_1492/ABUW_RS07250-ABUW_RS07275*). This operon encodes the proteins responsible for the synthesis of the Csu pilus, a type I chaperone-usher pilus involved in attachment and biofilm formation (39–41). Moreover, the *abaM*::T26 mutant also showed higher expression of some genes of the acinetin 505 biosynthetic operon, which has also been linked to biofilm formation in A. baumannii ATCC 17978 (42).

A previous comparison of the transcriptomes of the multidrug resistant clinical A. baumannii strain 863 with an isogenic *abaI* deletion mutant highlighted the differential regulation of 352 genes involved in carbon source metabolism, energy production, stress response, and translation (43). However, apart from *abaI*, no other common differentially regulated genes could be identified when the A5075 *abaI* and the 863 *abaI* mutant transcriptomes are compared. This may be because of the different strains and growth conditions and sampling times used. In addition, the *abaI* mutant reported by Ng et al. (43) exhibited a growth defect. This raises the possibility of a secondary mutation contributing to the transcriptome data, which was not validated by chemical or genetic complementation with OHC12 or *abaI*, respectively.

In the transcriptomic experiments presented here, the *abaI* transcripts were found in larger amounts in the *abaI* mutant in contrast to the *abaI*::*lux* promoter fusion data where, as expected, *abaI* expression was lower in the *abaI* mutant and stimulated by provision of exogenous OHC12. However, the regulation of A. baumannii QS is complex, especially given the nature of incoherent feed-forward loops, our lack of understanding of the mode of action of AbaM and the impact of other regulatory factors on the system. In addition, the promoter fusion assays were carried out over the entire growth curve in 96-well plates, whereas the RNA was prepared from cells at a single time point grown in larger volumes. In these experiments, the peak of *abaI* promoter activity was in mid/late log phase, whereas the RNA-seq samples were prepared from late stationary-phase cultures. It is also possible that the transposon insertion in *abaI* impacts the amount and stability of the transcript.

For *abaM*, we found that promoter activity and transcript levels mutant were similar. While the activity of the promoter increased by ∼50% in the *abaM* mutant compared to the wild type, we found a log_2_ fold change of ∼0.9 in the *abaM* mutant. This was just below the cutoff applied to our data (see Table S4). In the *abaI* mutant, *abaM* was not differentially expressed at the late stationary time point chosen for the RNA-seq. Consequently, future work will be required to unravel these observations with respect to *abaI* and *abaM* regulation, particularly in the context of growth environment.

Overall, the work described here establishes that AbaM plays a central role in regulating QS, surface motility, biofilm formation, and virulence. The apparently contradictory regulatory impact of *abaM* and *abaI* mutations that result in either increased or no AHL production, respectively, on the expression of genes such as the *csu* cluster can be explained as follows. In an *abaI* mutant (no AHLs), *abaM* expression is reduced and hence *csu* expression is increased. In an *abaM* mutant *csu* expression is also increased since AbaM is absent (Fig. 7). Further work will be required to elucidate the biochemical function and mechanism of action of AbaM and the RsaM protein family in general.

## MATERIALS AND METHODS

### Strains and growth conditions.

The strains and plasmids used are listed in Table S1 in the supplemental material. A. baumannii AB5075 (29) and the isogenic *abaI*::T26 and *abaM*::T26 mutants were obtained from the transposon library available from the University of Washington (44). A. baumannii was routinely grown in low-sodium chloride (5 g/liter) lysogeny broth (LS-LB). OHC12 was synthesized as described previously (45). The opaque and translucent phases of the wild type and mutants were separated as described by Tipton et al. (11) after growth on phase-observation LB (PO-LB) plates and observation of colonies under light microscopy using oblique indirect illumination.

### Construction of a genetically complemented *abaM*::T26 strain.

Plasmid pMQ557M (see Table S1) was obtained by digesting pMQ557 with PmlI (to remove the genes required for yeast replication) and religating the resulting large linear product. The *abaM* gene plus 768 bp from its upstream region (containing the predicted native promoter) were amplified by PCR using the primers listed in Table S2. The PCR fragments were digested with BamHI and KpnI and ligated in the multiple cloning site (MCS) of both pMQpMQ557M and introduced into *abaM*::T26 by electroporation. The stability of the vector pMQ557M and *abaM* complementing plasmid pMQ_*abaM* in both A5075 and the *abaM* mutant were confirmed by repeated daily subculture and plating out on LB agar with or without hygromycin (125 μg/ml) to determine viable counts as CFU/ml (see Fig. S6).

### Construction of *abaM*::*luxCDABE* and *abaI*::*luxCDABE* promoter fusions.

The *abaR* gene and the intergenic region between *abaR* and *abaM* (for the *abaM* fusion) or the region between *abaR* and the *abaI* (for the *abaI* fusion) were amplified by PCR and ligated in pGEM-T Easy using the pGEM-T Easy Vector System (Promega). The resulting plasmids and the promoterless *luxCDABE* operon were digested with KpnI and BamHI and ligated in order to introduce the *lux* operon downstream of the predicted promoter of *abaM* or *abaI*. These constructs were transferred into the MCS of the miniTn*7*T in pUC18T-miniTn*7*T-Hyg^R^ plasmid (see Table S1) after digestion with NotI and PstI and ligation of the corresponding fragments.

MiniTn*7*T-based constructs were inserted into A. baumannii through four-parental conjugation. Briefly, phosphate-buffered saline-washed overnight cultures of the Escherichia coli DH5α donor strain (containing pUC18T-mini-Tn*7*T_Hyg^R^_*abaR*_P*abaM*::*lux* or the pUC18T-mini-Tn*7*T_Hyg^R^_*abaR*_P*abaI*::*lux*), the E. coli DH5α helper strain (containing pUX-B13), the E. coli DH5α mobilizable strain (containing pRK600), and the A. baumannii recipient strain were mixed in a 1:1:1:1 ratio and grown on LB agar prior to counterselection with hygromycin (125 μg/ml for miniTn*7* selection) and gentamicin (100 μg/ml).

The miniTn*7* transposon (46) carrying the *abaM* promoter-*lux* operon fusion was inserted into A. baumannii AB5075 and the isogenic *abaI*::T26 and *abaM*::T26 mutants, whereas the *abaI* promoter-*lux* fusion was inserted into AB5075 and the isogenic *abaI*:T26 mutant. The bioluminescence output from the reporter fusions as a function of bacterial growth was quantified using an Infinite 200 PRO (Tecan Diagnostics) plate-reader over 24 h, and the optical density at 600 nm (OD_600_) and relative light units (RLUs) were recorded every 30 min. When required, OHC12 was added at 200 nM unless otherwise stated.

### Biofilm assays.

Strains to be tested were inoculated into 1.5-ml polypropylene microcentrifuge tubes in LS-LB with or without OHC12, followed by incubation under static conditions at 37°C for 24 h. Biofilms were quantified by staining with 0.25% crystal violet and extraction with ethanol, and the absorbance (*A*_585_) was recorded.

### Surface motility assays.

Surface motility was quantified as previously described (11) on LS-LB plates with or without OHC12 and containing 0.3% Eiken agar. Plates were incubated at 30°C for 16 h.

### AHL extraction and detection.

Cell-free supernatants from cultures grown in LS-LB under static conditions at 37°C were sterile filtered and extracted with acidified ethyl acetate. Extracts were evaporated to dryness and subjected to LC-MS/MS as previously described (45).

### *G. mellonella* killing assays.

G. mellonella larvae (Trularv) were obtained from BioSystems Technology, Ltd., Devon, United Kingdom. Assays were performed as described previously (11). Briefly, 2 × 10^4^ or 2 × 10^5^ CFU of Acinetobacter were injected into the larval hemolymph and incubated at 37°C, and the larvae were monitored for viability. At least 10 larvae were used for each strain and assay.

### Total RNA extraction and RNA-seq.

Bacteria were cultured in LS-LB under static conditions at 37°C for 18 h. The cells were resuspended in RNAprotect (Qiagen) prior to extracting total RNA using an RNeasy minikit (Qiagen). After treatment with DNA-free (Invitrogen), the absence of DNA contamination was confirmed using PCR, and the quality and quantity of the RNA samples was established using a 2100 Bioanalyzer (Agilent). Samples were sent for 150-bp paired-end sequencing via an Illumina platform and bioinformatic analysis to NovoGene (Hong Kong, China). 

### Quantitative real-time PCR.

Complementary DNA (cDNA) synthesis and qPCR were carried out using LunaScript RT Supermix and Luna Universal qPCR Master Mix (New England BioLabs), respectively. The oligonucleotides used for qPCR are listed in Table S2 and qPCR was carried out in triplicate using a 7500 real-time PCR system (Thermo Fisher). Negative controls lacking template or RNA incubated without reverse transcriptase were included. The housekeeping gene *rpoB* was used as endogenous control for normalization.

### Reverse transcription-PCR.

cDNA was amplified using Q5 high-fidelity polymerase (New England Biolabs) with specific primers annealing in the coding region of each gene. Genomic DNA, extracted with a DNeasy blood and tissue kit (Qiagen), was used as a positive control. The PCR products were run in a 1.5% agarose electrophoresis gel before imaging under UV light using a Gel Doc XR+ Imager (Bio-Rad).

### Data availability.

Bacterial sequencing data have been deposited in NCBI's Gene Expression Omnibus (47) and are accessible through GEO series accession number GSE151925.

## Supplementary Material

Supplemental file 1
