# Supplementary material for: AbaM Regulates Quorum Sensing, Biofilm Formation, and Virulence in Acinetobacter baumannii
Source: J Bacteriol. 2021 Mar 23;203(8):e00635-20. doi: 10.1128/JB.00635-20 (PMC8088503; doi:10.1128/JB.00635-20)
Supplement: Supplemental file 1 [file JB.00635-20-s0001.pdf]

SUPPLEMENTAL MATERIAL

Table S1: Strains and plasmids used in this study

**Strain or plasmid**	**Description**	**Reference or source**
**STRAINS**		
Escherichia coli		
E. coli K-12 DH5α	F^{-} *endA1 glnV44 thi-1 recA1 relA1 gyrA96 deoR nupG purB20* ϕ80d*lacZ*ΔM15 Δ(*lacZYA-argF*)U169 hsdR17($r_K^{-}m_K^{+}$) λ^{-}	Lab stock
Acinetobacter baumannii		
AB5075	Wild-type parental strain of the hypervirulent, multi-drug resistant *A. baumannii* AB5075, isolated from a patient with tibia osteomyelitis.	(Jacobs *et al*, 2014)
abaM::T26	AB5075 with the T26 transposon disrupting the *rsaM* orthologue *abaM* (*ABUW_3775*).	(Gallagher *et al,* 2015)
abaI::T26	AB5075 with the T26 transposon disrupting the quorum sensing synthase gene *abaI* (*ABUW_3776*).	(Gallagher *et al*, 2015)

AB5075 P*abaM*::*lux*	AB5075 carrying the mini-Tn*7*T_Hyg^{R}_P*abaM*::*lux* insertion.	This study
AB5075 P*abaI*::*lux*	AB5075 carrying the mini-Tn*7*T_Hyg^{R}_P*abaI*::*lux* insertion.	This study
abaM::T26 P*abaM*::*lux*	*abaM*::T26 carrying the mini-Tn*7*T_Hyg^{R}_P*abaM*::*lux* insertion.	This study
abaI::T26 P*abaM*::*lux*	*abaI*::T26 carrying the mini-Tn*7*T_Hyg^{R}_P*abaM*::*lux* insertion.	This study
abaI::T26 P*abaI*::*lux*	*abaI*::T26 carrying the mini-Tn*7*T_Hyg^{R}_P*abaI*::*lux* insertion.	This study
AB5075 pMQ557M	AB5075 carrying the pMQ557M plasmid.	This study
abaM::T26 pMQ557M	*abaM*::T26 carrying the pMQ557M plasmid.	This study.
abaM::T26 pMQ_*abaM*	*abaM*::T26 carrying the complementation plasmid pMQ_*abaM*.	This study
**PLASMIDS**		
pGEM®-T Easy	Commercial plasmid for PCR cloning.	Promega
pBluelux	pBlueScript II KS(+) (Agilent Technologies) plasmid containing the promoterless *luxCDABE* operon.	Lab stock

pBluescript-IISK_KO_Multi_HYG_UTRs	pBlueScript II KS(+) (Agilent Technologies) containing the hygromycin resistance cassette (*hph*).	Lab stock
pGEM_ *abaR*_P*abaM*::*lux*	pGEM®-T Easy containing *abaR* gene, the *abaR*-*abaM* intergenic region and the *abaM* promoter – *lux* operon fusion cloned in the MCS.	This study
pGEM_ *abaR*_P*abaI*::*lux*	pGEM®-T Easy containing the region from the *abaR* gene to the *abaI* promoter – *lux* operon fusion cloned in the MCS.	This study
pUC18T-mini-Tn*7*T	pUC18 plasmid with the mini-Tn*7* containing only terminator regions and a multi-cloning site (MCS).	(Choi *et al*, 2005)
pUC18T-miniTn*7*T_Hyg^R	pUC18T-mini-Tn*7*T with the *hph* cassette cloned in the MCS.	This study
pUC18T-mini-Tn*7*T_Hyg^R_*abaR*_P*abaM*::*lux*	pUC18T-mini-Tn*7*T_Hyg^R with the *abaR* gene, the *abaR*-*abaM* intergenic region and the *abaM* promoter – *lux* operon fusion cloned in the MCS.	This study
pUC18T-mini-Tn*7*T_Hyg^R_*abaR*_P*abaI*::*lux*	pUC18T-mini-Tn*7*T_Hyg^R with the region from the *abaR* gene to the *abaI* promoter – *lux* operon fusion cloned in the MCS.	

pUX-B13	Plasmid carrying the transposase genes required for four-parental conjugation.	Lab stock
pRK600	Helper plasmid carrying the mobilization genes required for four-parental conjugation.	Lab stock
pMQ557M	pMQ557 vector, replicative and stable in *A. baumannii* containing the *hph* cassette as antibiotic marker, with the yeast replication genes removed.	Robert Shanks (University of Pittsburgh)
pMQ_*abaM*	pMQ557M containing the *abaM* gene and 758 bp of the upstream region.	This study.

References

Jacobs AC, Thompson MG, Black CC, Kessler JL, Clark LP, McQueary CN, Gancz HY, Corey BW, Moon JK, Si Y, Owen MT, Hallock JD, Kwak YI, Summers A, Li CZ, Rasko DA, Penwell WF, Honnold CL, Wise MC, Waterman PE, Lesho EP, Stewart RL, Actis LA, Palys TJ, Craft DW, Zurawski D V. (2014). AB5075, a highly virulent isolate of *Acinetobacter baumannii*, as a model strain for the evaluation of pathogenesis and antimicrobial treatments. *MBio*. 5:e01076-14.

Gallagher LA, Ramage E, Weiss EJ, Radey M, Hayden HS, Held KG, Huse HK, Zurawski D V, Brittnacher MJ, Manoil C. (2015). Resources for genetic and genomic analysis of emerging pathogen *Acinetobacter baumannii*. *J Bacteriol* 197:2027–35.

Choi K-H, Gaynor JB, White KG, Lopez C, Bosio CM, Karkhoff-Schweizer RR, Schweizer HP. (2005). A Tn*7*-based broad-range bacterial cloning and expression system. *Nat Methods* 2:443–448.

Table S2: Primers used in this study. Bases corresponding to the restriction sites are underlined.

Primer	Sequence (5’ → 3’)	Description
P*abaM*::*lux*_FW	CTCCGATTAATTATAATTAACC	Construction of the P*abaM*::*lux* and P*abaI*::*lux* reporters
P*abaM*::*lux*_RV	GCGCGGATCCGCGCGGTACCCCATGCTACCTGCTTAAGTACCC	Construction of the P*abaM*::*lux* reporter
P*abaI*::*lux*_RV	GGATCCGGTACCCATTACAAGTGCTTCCACTTA	Construction of the P*abaI*::*lux* reporter
*rpoB*_qPCR_FW	TCGTGTTGAGCGTGCTGTTA	qPCR of the endogenous control gene *rpoB*
*rpoB*_qPCR_RV	TGCAGCAGCAACTGGTTTTG	qPCR of the endogenous control gene *rpoB*
*csuAB*_qPCR_FW	AGCAGCAACAGGTGGCAATA	qPCR of the *csuA/B* gene
*csuAB*_qPCR_RV	GGTCTGTACGTTCACCACCAT	qPCR of the *csuA/B* gene
*3773*_qPCR_FW	AGTGTCACTGCGGGTTACTG	qPCR of the *ABUW_3773* gene
*3773*_qPCR_RV	CTAGGTTGTCCCGCCTCATC	qPCR of the *ABUW_3773* gene
Hyg_FW	TCATCACTGCAGATGAAAAAGCCTGAACTCACC	Cloning of *hph* cassette in mini-Tn7T.

Hyg_RV	TCATCACTGCAGGGGGGATCGATCCCGGTCGG	Cloning of *hph* cassette in mini-Tn7T.
*abaM*_compl_FW_*Bam*HI	ATATGGATCCTTGCTCTCATTAGACTCCATTCAC	*abaM*::T26 complementation.
*abaM*_compl_RV_*Kpn*I	ATATGGTACCGTGCTTCCACTTATTTTTCAAGT	*abaM*::T26 complementation.
*abaR*_RV	CTACAAAAGCCCTAGCATTACAGC	RT-PCRs (*abaR-abaM*)
*abaM*_FW	GGTTAGCATACCCCCTCATTTC	RT-PCRs (*abaM* and *abaM-abaI*)
*abaM*_RV	GGTTTGACTTAATGAAGACTCG	RT-PCRs (*abaM* and *abaR-abaM*)
*abaI*_FW	CAATTTTTCAGAAGGCCTATATACC	RT-PCRs (*abaI)*
*abaI*_RV	CAATCAAGCATGCAAACATC	RT-PCRs (*abaI* and *abaM-abaI*)

Table S3: Differentially expressed genes (log$_2$(fold change) $\geq$ 1) in *abaI*::T26 (compared with AB5075 wild-type).

**Gene id**	**Log$_2$(Fold change)**	**Description**	**NCBI Protein Accession**
ABUW_RS18385	4.3142	*N*-acylhomoserine lactone synthase (*abaI*)	WP_001020940.1
ABUW_RS07260	4.2853	SCPU domain-containing protein (*csuB*)	WP_000876475.1

ABUW_RS07255	4.1072	protein CsuA (*csuA*)	WP_000577009.1
ABUW_RS07265	3.9262	molecular chaperone (*csuC*)	WP_001065473.1
ABUW_RS07250	3.9062	SCPU domain-containing protein (*csuA/B*)	WP_000790104.1
ABUW_RS07275	3.692	protein CsuE (*csuE*)	WP_002017500.1
ABUW_RS07270	3.6287	fimbrial biogenesis outer membrane usher protein (*csuD*)	WP_000603294.1
ABUW_RS07245	2.8129	TetR/AcrR family transcriptional regulator	WP_000590096.1
ABUW_RS07925	1.9432	alcohol dehydrogenase	WP_000874704.1
ABUW_RS01970	1.9355	flavohemoprotein	WP_000188888.1
ABUW_RS07910	1.9263	aldehyde dehydrogenase	WP_001269058.1
ABUW_RS07630	1.8189	hypothetical protein	WP_000783724.1
ABUW_RS07640	1.7361	hypothetical protein	WP_000772607.1
ABUW_RS07620	1.5361	hypothetical protein	WP_000020771.1
ABUW_RS07905	1.5245	ethanolamine permease	WP_001075439.1
ABUW_RS07625	1.4155	LLM class flavin-dependent oxidoreductase	WP_001257199.1
ABUW_RS07240	1.4129	hypothetical protein	WP_000637306.1
ABUW_RS08275	1.402	DNA methylase	WP_000652017.1
ABUW_RS06205	1.3599	DNA helicase	WP_000106166.1
ABUW_RS07280	1.3354	hypothetical protein	WP_000821199.1
ABUW_RS08025	1.3233	hypothetical protein	WP_001092426.1
ABUW_RS07895	1.3225	ethanolamine ammonia-lyase subunit EutC	WP_000774022.1

ABUW_RS19740	1.3116	hypothetical protein	WP_002017532.1
ABUW_RS08010	1.3043	hypothetical protein	WP_001243508.1
ABUW_RS07980	1.2832	glutathione S-transferase family protein	WP_001279985.1
ABUW_RS07800	1.2821	membrane protein	WP_001228338.1
ABUW_RS08160	1.2796	3-oxoacyl-ACP reductase	WP_000132115.1
ABUW_RS07445	1.2788	dicarboxylate/amino acid:cation symporter	WP_000347180.1
ABUW_RS08155	1.2734	acetyl-CoA C-acyltransferase	WP_000047940.1
ABUW_RS05005	1.2604	alpha/beta hydrolase	WP_000149921.1
ABUW_RS08165	1.2485	hypothetical protein	WP_001097352.1
ABUW_RS08145	1.2425	LysR family transcriptional regulator	WP_001024733.1
ABUW_RS19150	1.2359	hypothetical protein	WP_001024502.1
ABUW_RS11580	1.2324	taurine ABC transporter permease	WP_001092656.1
ABUW_RS07985	1.2211	short-chain dehydrogenase	WP_000135428.1
ABUW_RS08190	1.2166	phospholipase C%2C phosphocholine-specific	WP_000633003.1
ABUW_RS07715	1.2132	2-amino-4-hydroxy-6-hydroxymethyldihydropteridine pyrophosphokinase	WP_000993407.1
ABUW_RS08170	1.2116	esterase	WP_000588779.1
ABUW_RS19155	1.2107	hypothetical protein	WP_000241707.1

ABUW_RS07725	1.2067	K(+)-transporting ATPase subunit B	WP_001097170.1
ABUW_RS07435	1.186	MFS transporter	WP_001066110.1
ABUW_RS11590	1.1859	taurine ABC transporter substrate-binding protein	WP_000105187.1
ABUW_RS19145	1.1712	transcriptional regulator	WP_000096600.1
ABUW_RS07920	1.1707	alpha/beta hydrolase	WP_001098135.1
ABUW_RS07955	1.1695	aminopeptidase N	WP_001017234.1
ABUW_RS05010	1.1572	sulfate ABC transporter substrate-binding protein	WP_001212600.1
ABUW_RS07845	1.1519	tRNA (adenosine(37)-N6)-threonylcarbamoyltransferase complex ATPase subunit type 1 TsaE	WP_031944287.1
ABUW_RS08125	1.1426	membrane protein	WP_001169538.1
ABUW_RS07465	1.1379	methionine ABC transporter ATP-binding protein	WP_000614025.1
ABUW_RS19770	1.1377	hypothetical protein	-
ABUW_RS08255	1.132	hypothetical protein	WP_001238662.1
ABUW_RS19165	1.1273	toxic anion resistance protein	WP_000933769.1
ABUW_RS07360	1.123	acyl-CoA desaturase	WP_000032710.1
ABUW_RS19170	1.1197	hypothetical protein	WP_001093570.1
ABUW_RS07935	1.1175	5-carboxymethyl-2-hydroxymuconate isomerase	WP_001120984.1
ABUW_RS07460	1.1162	ABC transporter permease	WP_001205190.1

ABUW_RS07505	1.1138	tRNA uridine-5-carboxymethylaminomethyl(34) synthesis enzyme MnmG	WP_000559187.1
ABUW_RS08225	1.1107	alpha/beta hydrolase	WP_001290021.1
ABUW_RS19105	1.1103	hypothetical protein	WP_001070890.1
ABUW_RS19075	1.1051	hypothetical protein	WP_001012847.1
ABUW_RS19080	1.1038	thermonuclease	WP_000861205.1
ABUW_RS07735	1.1031	sensor histidine kinase KdpD	WP_001191611.1
ABUW_RS07660	1.0966	peptide chain release factor N(5)-glutamine methyltransferase	WP_001017509.1
ABUW_RS07605	1.0929	poly-beta-1%2C6-N-acetyl-D-glucosamine N-deacetylase PgaB	WP_001061302.1
ABUW_RS07775	1.0919	hypothetical protein	WP_000756505.1
ABUW_RS07650	1.0916	type 1 glutamine amidotransferase domain-containing protein	WP_000735895.1
ABUW_RS07395	1.0911	DNA-binding response regulator	WP_000526534.1
ABUW_RS08035	1.0891	TonB-dependent siderophore receptor	WP_000527015.1
ABUW_RS07720	1.0799	potassium-transporting ATPase subunit KdpA	WP_000891202.1

ABUW_RS07305	1.0773	TetR/AcrR family transcriptional regulator	WP_000792929.1
ABUW_RS19160	1.0754	hypothetical protein	WP_000661588.1
ABUW_RS08095	1.0722	universal stress protein	WP_001109442.1
ABUW_RS07785	1.0593	hypothetical protein	WP_000920881.1
ABUW_RS08250	1.0587	MFS transporter	WP_071543520.1
ABUW_RS08235	1.0544	PepSY domain-containing protein	WP_000075461.1
ABUW_RS08280	1.0528	nitrate transporter	WP_000039924.1
ABUW_RS07235	1.052	FadR family transcriptional regulator	WP_000572518.1
ABUW_RS07840	1.05	pseudouridylate synthase	WP_000097958.1
ABUW_RS07990	1.0454	oxidoreductase	WP_000920714.1
ABUW_RS08110	1.0389	RDD family protein	WP_000221476.1
ABUW_RS07710	1.0383	dihydroneopterin aldolase	WP_000338779.1
ABUW_RS07600	1.0353	poly-beta-1%2C6 N-acetyl-D-glucosamine export porin PgaA	WP_000913301.1
ABUW_RS07560	1.035	protoheme IX farnesyltransferase	WP_000915319.1
ABUW_RS19300	1.0227	aminoglycoside O-phosphotransferase APH(3'')-Ib	WP_025464697.1
ABUW_RS07930	1.0224	helicase	WP_000808297.1
ABUW_RS08090	1.0174	ABC transporter ATP-binding protein	WP_000193596.1

ABUW_RS08185	1.0159	DNA polymerase III subunit gamma/tau	WP_045887671.1
ABUW_RS07815	0.99981	DUF441 domain-containing protein	WP_000880863.1
ABUW_RS15330	-1.016	hypothetical protein	WP_001001666.1
ABUW_RS09690	-1.0661	zonular occludens toxin	WP_032017212.1
ABUW_RS13220	-1.0776	hypothetical protein	WP_001037898.1
ABUW_RS15280	-1.1274	hypothetical protein	WP_000206132.1
ABUW_RS09695	-1.1693	hypothetical protein	WP_032017213.1
ABUW_RS01270	-1.1982	sulfate permease	WP_001111063.1
ABUW_RS09700	-1.2079	hypothetical protein	WP_032017215.1
ABUW_RS01160	-1.2284	aquaporin Z	WP_001045986.1
ABUW_RS12990	-1.4531	hypothetical protein	WP_001034729.1

Table S4: Differentially expressed genes (log_2(fold change) ≥ 1) in *abaM*::T26 (compared with AB5075 wild-type)

**Gene id**	**Log_2(Fold change)**	**Description**	**NCBI Protein Accession**
ABUW_RS07245	2.9052	TetR/AcrR family transcriptional regulator	WP_000590096.1
ABUW_RS07260	2.878	SCPU domain-containing protein (*csuB*)	WP_000876475.1
ABUW_RS18365	2.8531	acyl-CoA dehydrogenase (*ABUW_3772*) Ac-505 biosynthetic operon	WP_000060267.1
ABUW_RS11870	2.7441	hypothetical protein	WP_000108365.1

ABUW_RS18350	2.6991	RND transporter (*ABUW_3769*). Ac-505 biosynthetic operon	WP_000256252.1
ABUW_RS18355	2.6293	non-ribosomal peptide synthetase (ABUW_3770)	WP_001060991.1
ABUW_RS18375	2.554	LuxR family transcriptional regulator (*abaR*)	WP_000446790.1
ABUW_RS07265	2.5191	molecular chaperone (*csuC*)	WP_001065473.1
ABUW_RS07250	2.4684	SCPU domain-containing protein (*csuA/B*)	WP_000790104.1
ABUW_RS18385	2.4087	N-acylhomoserine lactone synthase (*abaI*)	WP_001020940.1
ABUW_RS18370	2.3341	acyl-CoA synthetase (*ABUW_3773*) Ac-505 biosynthetic operon	WP_000279948.1
ABUW_RS07255	2.323	protein CsuA (*csuA*)	WP_000577009.1
ABUW_RS07270	2.2652	fimbrial biogenesis outer membrane usher protein (*csuD*)	WP_000603294.1
ABUW_RS07275	2.1508	protein CsuE (*csuE*)	WP_002017500.1
ABUW_RS07145	1.8615	DUF2171 domain-containing protein	WP_001094391.1
ABUW_RS11840	1.7989	stress-induced protein	WP_000024222.1
ABUW_RS08050	1.7574	hypothetical protein	WP_001123841.1
ABUW_RS11875	1.7165	hypothetical protein	WP_000132046.1
ABUW_RS19150	1.5586	hypothetical protein	WP_001024502.1
ABUW_RS19060	1.4374	hypothetical protein	WP_000910917.1
ABUW_RS19165	1.4373	toxic anion resistance protein	WP_000933769.1
ABUW_RS19145	1.4181	transcriptional regulator	WP_000096600.1
ABUW_RS03420	1.4179	tRNA-Asp	-
ABUW_RS01970	1.4088	flavohemoprotein	WP_000188888.1

ABUW_RS19155	1.3734	hypothetical protein	WP_000241707.1
ABUW_RS19115	1.3711	hypothetical protein	WP_001057663.1
ABUW_RS19170	1.3708	hypothetical protein	WP_001093570.1
ABUW_RS19140	1.3556	ParA family protein	WP_000724905.1
ABUW_RS13115	1.3406	hypothetical protein	WP_000980495.1
ABUW_RS19080	1.2162	thermonuclease	WP_000861205.1
ABUW_RS19240	1.202	hypothetical protein	-
ABUW_RS19305	1.1854	ANT(3'')-Ia family aminoglycoside nucleotidyltransferase AadA2	WP_001261740.1
ABUW_RS20015	1.1825	hypothetical protein	-
ABUW_RS19065	1.1742	hypothetical protein	WP_000095825.1
ABUW_RS12220	1.1605	hypothetical protein	WP_000008105.1
ABUW_RS19105	1.1574	hypothetical protein	WP_001070890.1
ABUW_RS11885	1.1476	hypothetical protein	WP_001136759.1
ABUW_RS13925	1.1447	23S rRNA (pseudouridine(1915)-N(3))-methyltransferase RlmH	WP_000702193.1
ABUW_RS20020	1.1374	transposition protein TniB	WP_001381192.1
ABUW_RS19595	1.0943	DNA-binding protein	WP_001096616.1
ABUW_RS19110	1.0805	DUF2786 domain-containing protein	WP_000389915.1
ABUW_RS19200	1.0625	hypothetical protein	WP_000790084.1
ABUW_RS19820	1.0579	hypothetical protein	-
ABUW_RS19190	1.0498	hypothetical protein	WP_000701003.1
ABUW_RS19235	1.0446	hypothetical protein	WP_000654348.1
ABUW_RS19180	1.0398	hypothetical protein	WP_000443897.1
ABUW_RS10070	1.0279	Fur family transcriptional regulator	WP_000207886.1

ABUW_RS13010	1.0165	DUF4142 domain-containing protein	WP_000644336.1
ABUW_RS19345	1.0122	molecular chaperone DnaJ	WP_000758689.1
ABUW_RS08580	1.009	DUF159 family protein	WP_000332547.1
ABUW_RS19300	1.0071	aminoglycoside O-phosphotransferase APH(3'')-Ib	WP_025464697.1
ABUW_RS11975	1.003	PaaI family thioesterase	WP_000445634.1
ABUW_RS10685	-0.99885	HU family DNA-binding protein	WP_001043034.1
ABUW_RS14775	-1.0208	outer membrane protein	WP_001202415.1
ABUW_RS17210	-1.0241	Na+/H+ antiporter subunit E	WP_001177202.1
ABUW_RS18725	-1.045	thiol:disulfide interchange protein DsbA/DsbL	WP_000737570.1
ABUW_RS15925	-1.0539	integration host factor subunit alpha	WP_000126166.1
ABUW_RS03825	-1.0607	hypothetical protein	WP_001278744.1
ABUW_RS15280	-1.0657	hypothetical protein	WP_000206132.1
ABUW_RS18190	-1.0826	ATP synthase subunit C	WP_000424060.1
ABUW_RS08725	-1.0884	hypothetical protein	WP_000179560.1
ABUW_RS02765	-1.0913	carbapenem-hydrolyzing class D beta-lactamase OXA-23	WP_001046004.1
ABUW_RS03465	-1.0935	alanine:cation symporter family protein	WP_001005337.1
ABUW_RS09695	-1.0973	hypothetical protein	WP_032017213.1
ABUW_RS17205	-1.1655	monovalent cation/H+ antiporter subunit D	WP_000459267.1
ABUW_RS04295	-1.1868	succinyl-CoA ligase subunit beta	WP_001048573.1
ABUW_RS01270	-1.2922	sulfate permease	WP_001111063.1
ABUW_RS13140	-1.3221	heavy metal-associated domain protein	WP_000770719.1

ABUW_RS14740	-1.3499	hemerythrin	WP_000782976.1
ABUW_RS15385	-1.35	NADH-quinone oxidoreductase subunit K	WP_000529822.1
ABUW_RS17440	-1.4553	membrane protein	WP_000472947.1
ABUW_RS03380	-1.5003	entericidin%2C EcnA/B family	WP_000757214.1
ABUW_RS17200	-1.5374	Na+/H+ antiporter subunit C	WP_000624229.1
ABUW_RS02775	-1.8507	phage tail assembly protein	WP_001071615.1
ABUW_RS12990	-2.1063	hypothetical protein	WP_001034729.1
ABUW_RS00585	-2.1307	hypothetical protein	WP_000770763.1

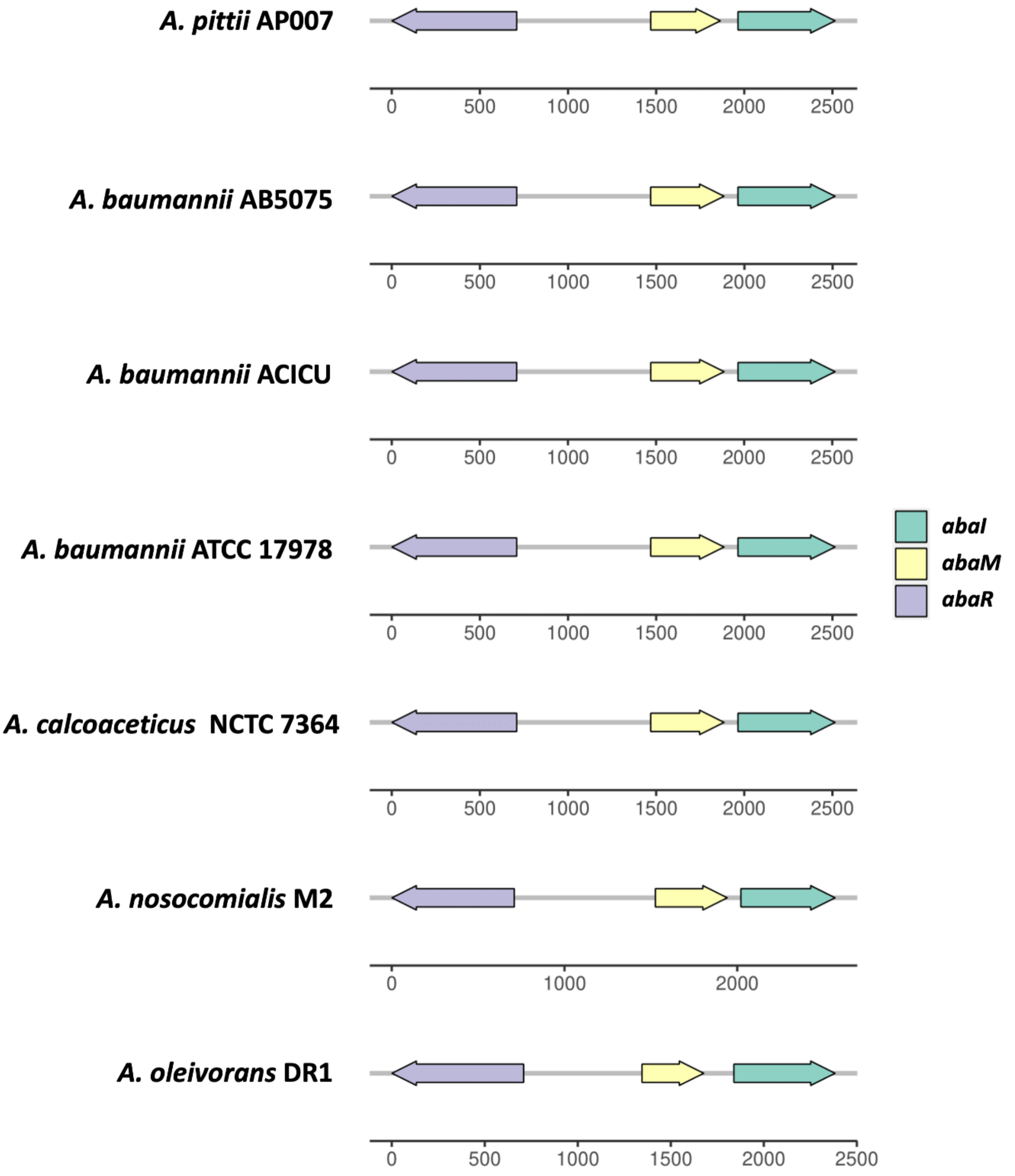

FIG S1 Conservation of quorum sensing locus organization among different *Acinetobacter spp.*

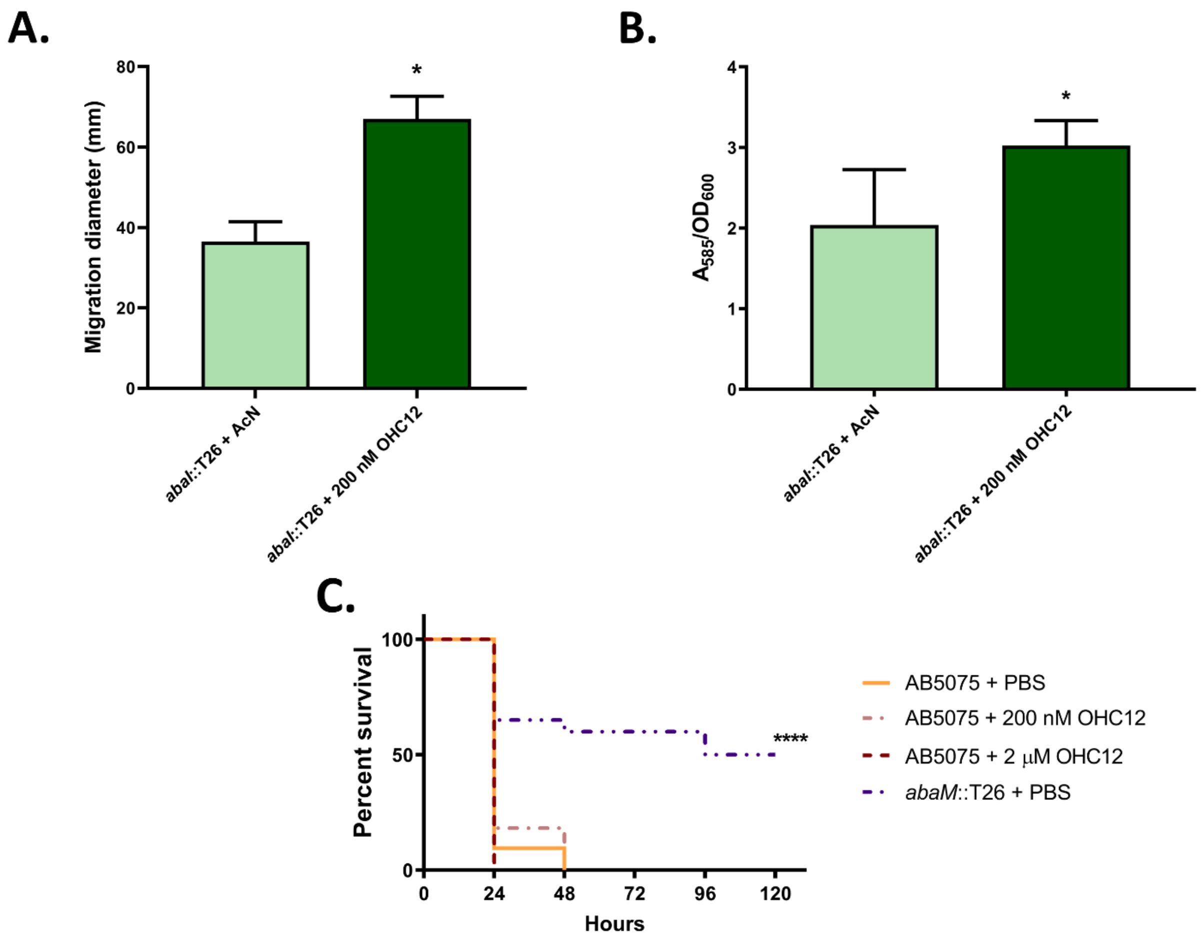

FIG S2 Contribution of OHC12 to AB5075 phenotypes. **(A)** Surface motility and (**B**) biofilm formation on polypropylene by the *abaI* mutant with and without OHC12. Acn, Acetonitrile solvent control. **(C)** *Galleria mellonella* larvae killing by wild type with and without OHC12 compared with the *abaM* mutant after inoculation of approximately 2 x 10^4 CFU/larvae. Asterisks indicate statistically significant differences compared to the wild-type AB5075 strain: **, $p \leq 0.01$; ***, $p \leq 0.001$; ****; $p \leq 0.0001$

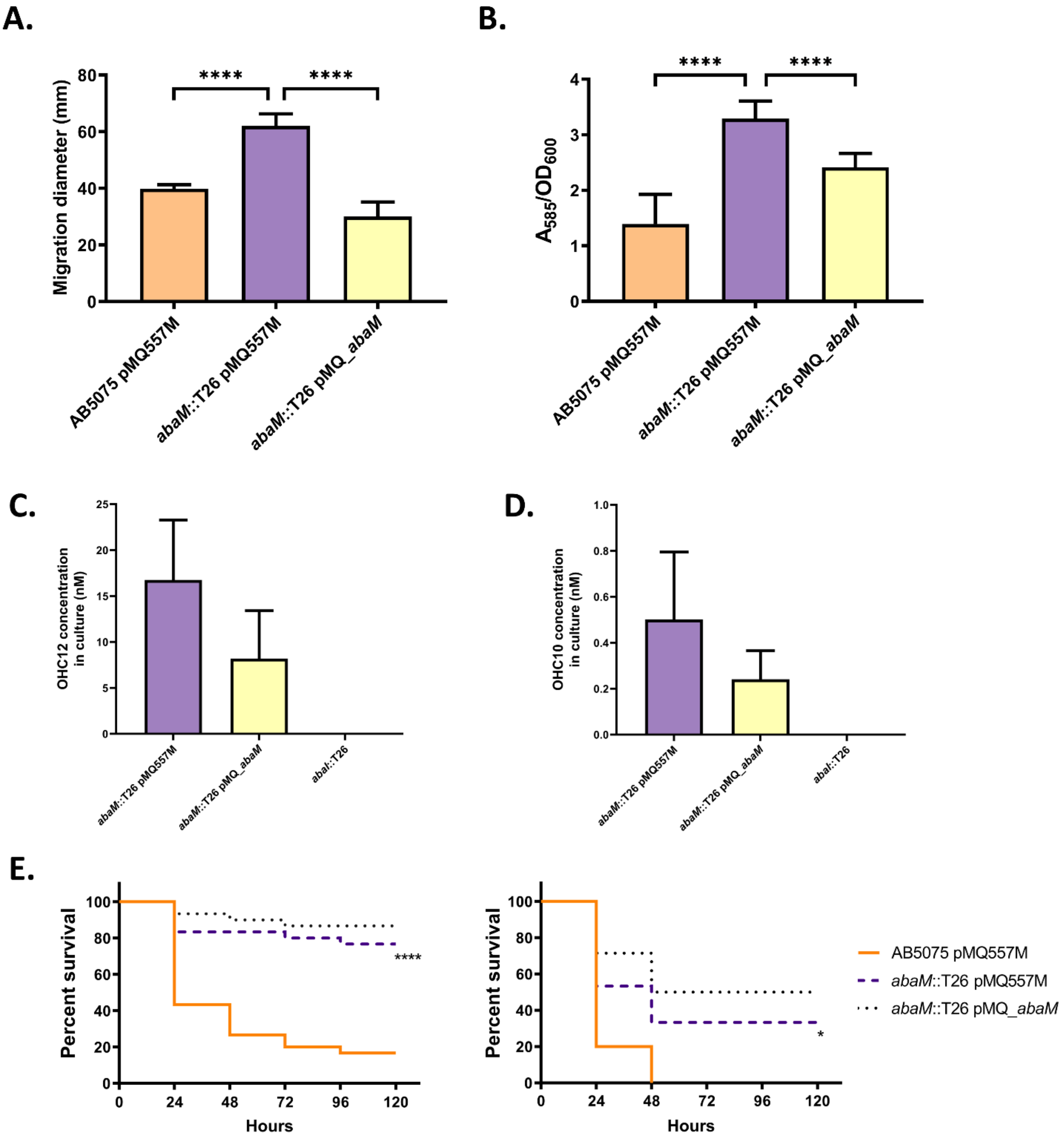

FIG S3 Genetic complementation of the *abaM*::T26 mutant. **(A)** Surface motility in 0.3% Eiken agar LS-LB plates. **(B)** Biofilm formation in polypropylene. **(C)** OHC12 production. **(D)** OHC10 production. **(E)** *Galleria mellonella* larvae killing after inoculation of approximately 2 x 10^4 (left) or 2 x 10^5 (right) CFU/larva. Asterisks indicate statistically significant differences compared with the wild-type AB5075 strain: *, p $\leq$ 0.05; ****, p $\leq$ 0.0001.

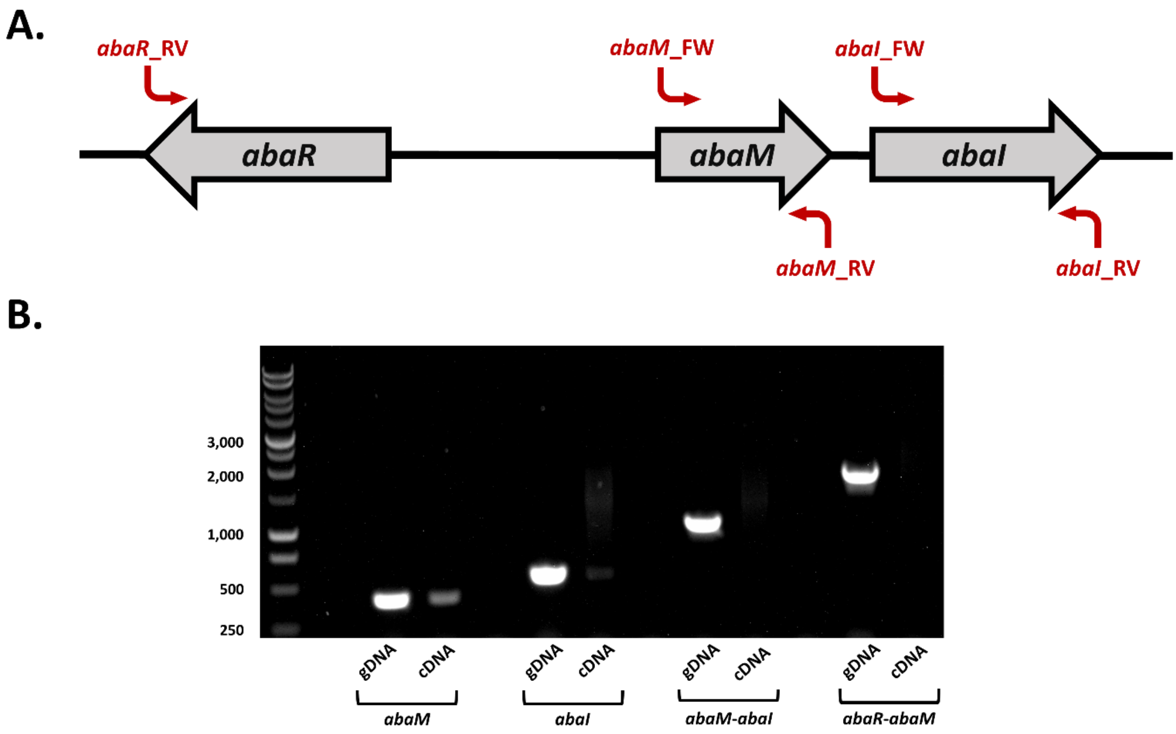

Fig. S4 The *abaM* and *abaI* genes are not co-transcribed. **(A)** Organization of the *abaRMI* region and the positions of the PCR primers used **(B)** Agarose gel showing single *abaM* and *abaI* transcripts. No cDNAs were obtained for *abaM-abaI* or for *abaR-abaM*. The left hand lane shows the DNA molecular markers. gDNA, genomic DNA control. The *abaM*_FW and *abaM*_RV primer pairs were used to amplify the *abaM* gene; *abaI*_FW and *abaI*_RV pair for the *abaI* gene, the *abaM*_FW and *abaI*_RV pair for the *abaM-abaI* region and the *abaR*_RV and *abaM*_RV pair for *abaR-abaM*.

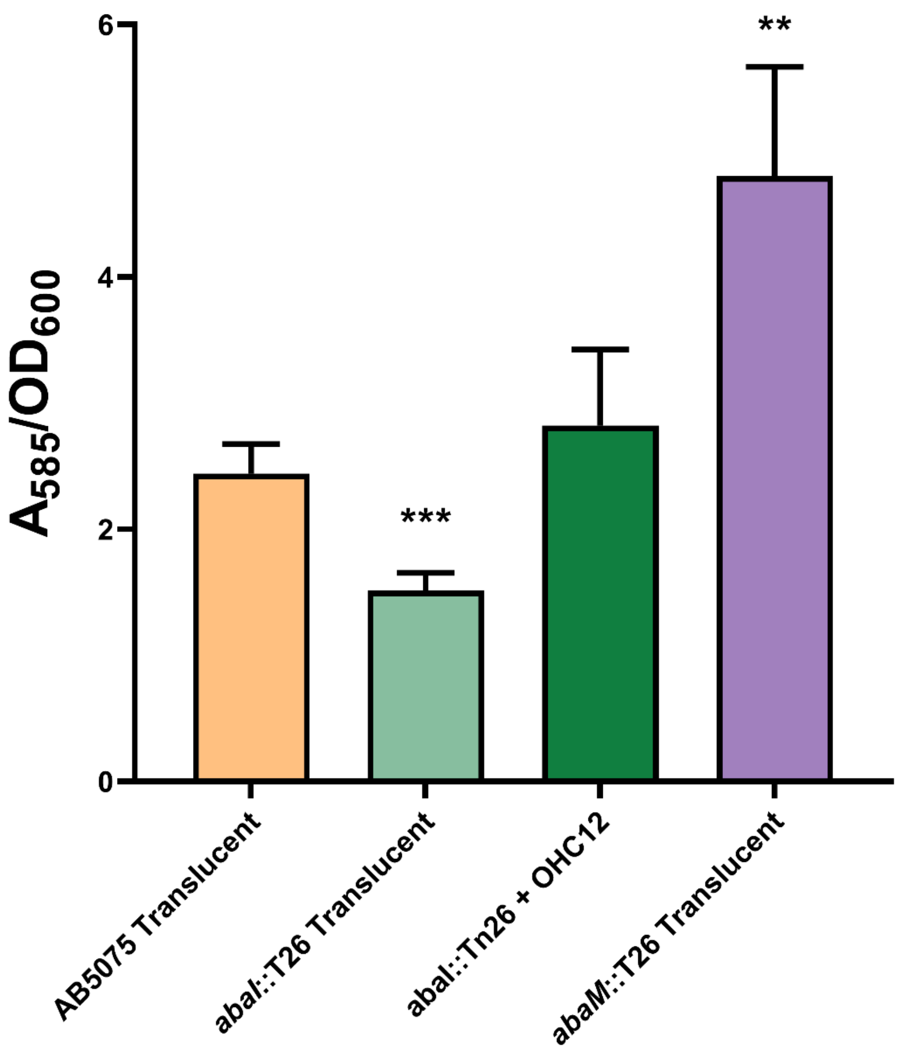

FIG S5 Biofilm formation on polypropylene tubes by translucent variants of AB5075 wild-type, *abaI*::T26, and *abaM*::T26. The *abaI*::T26 mutant was also supplemented with OHC12 (200 nM). Asterisks indicate statistically significant differences compared to the wild-type AB5075 strain: **, p ≤ 0.01; ****; p ≤ 0.0001.

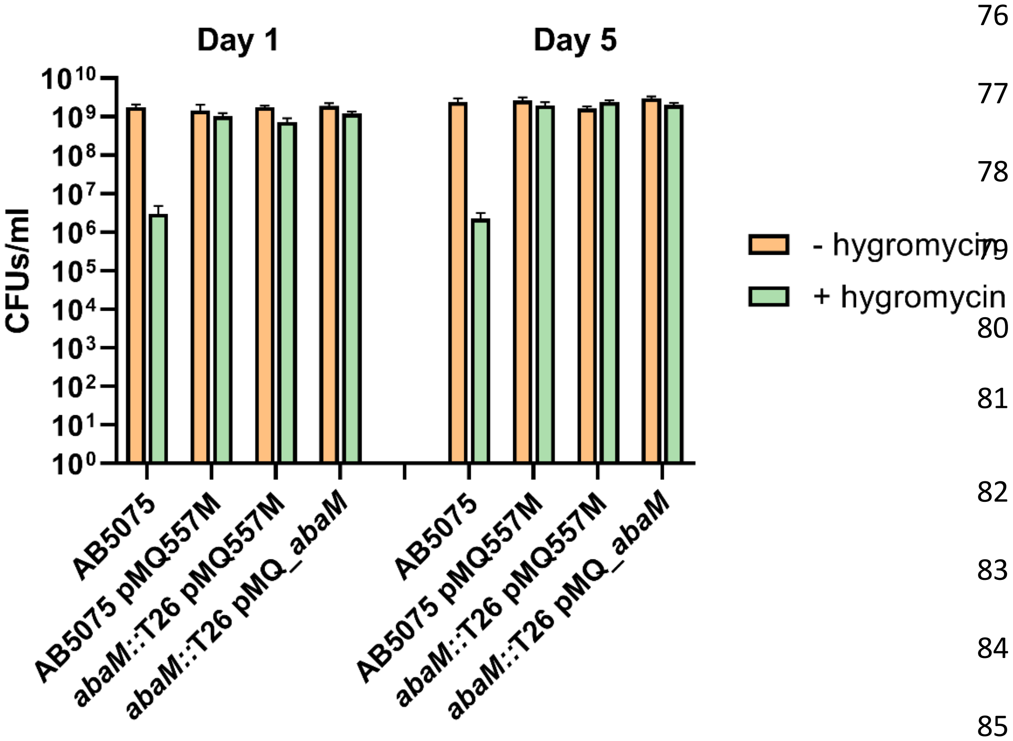

FIG S6 Stability of the vector pMQ557M and *abaM* complementing plasmid pMQabaM in the wild type AB5075 and *abaM* mutant in the presence and absence of hygromycin selection after 5 days of repeat daily subculturing.
